# Experimental selection reveals a trade-off between fecundity and lifespan in the coliphage Qß

**DOI:** 10.1098/rsob.130043

**Published:** 2013-06

**Authors:** Libertad García-Villada, John W. Drake

**Affiliations:** Laboratory of Molecular Genetics, National Institute of Environmental Health Sciences, National Institutes of Health, Research Triangle Park, NC 27709, USA

**Keywords:** phage life-history evolution, phage fecundity/lifespan trade-off, phage evolutionary constraints, bacteriophage Qß

## Abstract

Understanding virus evolution is key for improving ways to counteract virus-borne diseases. Results from comparative analyses have previously suggested a trade-off between fecundity and lifespan for viruses that infect the bacterium *Escherichia coli* (i.e. for coliphages), which, if confirmed, would define a particular constraint on the evolution of virus fecundity. Here, the occurrence of such a trade-off is investigated through a selection experiment using the coliphage Qß. Selection was applied for increased fecundity in three independent wild-type Qß populations, and the ability of the virions to remain viable outside the host was determined. The Qß life-history traits involved in the evolution of fecundity and the genetic changes associated with this evolution were also investigated. The results reveal that short-term evolution of increased fecundity in Qß was associated with decreased viability of phage virions. This trade-off apparently arose because fecundity increased at the expense of reducing the amount of resources (mainly time) invested per produced virion. Thus, the results also indicate that Qß fecundity may be enhanced through increases in the rates of adsorption to the host and progeny production. Finally, genomic sequencing of the evolved populations pinpointed sequences likely to be involved in the evolution of Qß fecundity.

## 2. Introduction

Life-history traits are demographics that shape the growth of populations. The principal life-history traits are age distribution of reproduction, fecundity and lifespan. Driven by natural selection, life-history traits are expected to evolve for all organisms to maximize their growth rates [1]. That different species show, for instance, different age distributions of reproduction, and that organisms have limited fecundities and lifespans, denote the existence of constraints on the evolution of life-history traits. These constraints may be classified into three overlapping categories: (i) phylogenetic, resulting from each lineage's particular evolutionary history; (ii) genetic, due to either a lack of genetic variation or to pleiotropy (multiple effects of a particular allele); and (iii) physiological, or trade-offs, due to differential allocation of an individual's resources to different physiological functions (reviewed in [2]). Trade-offs between fecundity and other components of fitness have been demonstrated for a number of species (reviewed in [3]) and indicate, unsurprisingly, that reproduction is costly.

The lytic life cycle of a phage (a virus that infects a bacterium) may be divided into six successive phases: (i) dispersal period, the time between consecutive infections; (ii) adsorption to the host cell; (iii) host-cell invasion, or infection *sensu stricto*; (iv) phage genome expression and replication; (v) assembly of phage progeny, or maturation; and (vi) lysis of the host cell with release of the phage progeny. The rates and timing of the processes underlying these phases characterize a phage's life-history traits [4], and the analysis of these traits and of their evolutionary constraints is key to understanding virus evolution [5].

A comparative study of the evolution of viral life histories [6] revealed a strongly positive correlation between the rates of multiplication and mortality across a wide variety of coliphages (phages whose natural host is the bacterium *Escherichia coli*). The multiplication rate of a phage is determined by the ratio of the average burst size (the number of progeny produced during the infection of a single cell) to the average latent period (the time between host infection and lysis), and it is therefore a useful measure of phage fecundity. The mortality rate of a phage, on the other hand, defines how rapidly a phage population loses its capability to generate infection. Thus, a positive correlation between these two traits suggests a trade-off between fecundity and lifespan. The predicted trade-off implies: (i) the existence of a physiological constraint on the evolution of coliphage fecundity and (ii) that coliphages, similar to other, more complex organisms, have also evolved life-history strategies to cope with evolutionary constraints through differential allocation of the host's resources to different phage life-cycle functions. The authors' analysis of possible causes of such a trade-off and their experimental results suggested that the lesser the relative amounts of time and energy invested in the production of phage virions, the greater the mortality rate [6].

Unfortunately, a correlation between life-history traits across different lineages, such as between fecundity and lifespan in coliphages, does not demonstrate a trade-off allocation between them because such correlations may result from the effects of other related traits that differ among the analysed lineages [7]. Thus, a more appropriate way to test the existence of a trade-off between different life-history traits is by imposing selection on one trait and determining whether there are (or are not) correlated changes in the other considered traits (reviewed in [2]).

Here, we used the model single-stranded RNA (ssRNA) coliphage Qß to test for an anticipated trade-off between fecundity and lifespan in coliphages through a selection experiment. First, three independent wild-type (wt) Qß populations were adapted to display an increased multiplication rate. Next, their capability to survive outside the host cell was determined and compared with that of the ancestral wt population. We further investigated which Qß life-history traits were involved in the evolution of the multiplication rate, together with the genetic changes associated with the observed phenotypic changes.

## 3. Results and discussion

### 3.1. Selection for increased Qß multiplication rate through *infectio interruptus*

The first objective of this study was to obtain, through selection, a Qß population displaying a multiplication rate greater than that of its ancestral wt population. Multiplication rate (*M*) is a measure of phage fecundity calculated as *M* = (burst size)/(latent period) scaled as phages/h. *M* may be increased by selecting for increased burst size and/or shortened latent period. Previous reports have shown that both burst size and latent period are susceptible to change through selection [8–10]. These traits are shaped by the processes that take place during phage lytic growth: (i) adsorption; (ii) infection; (iii) progeny production; and (iv) lysis of the infected host cell. Because there is an indirect connection between progeny maturation and the timing of lysis [11], we anticipated that the most effective approach for increasing *M* would be to select for variants displaying an increased rate of progeny production. In a synchronously growing phage population, such variants would be expected to accumulate their progeny more rapidly than their wt counterparts, so that their numbers of progeny would exceed that of the wild-type at any time before lysis (but keeping in mind that final differences in burst size between the variants and the wild-type are also expected to depend on the length of the latent period).

**Figure 1.**
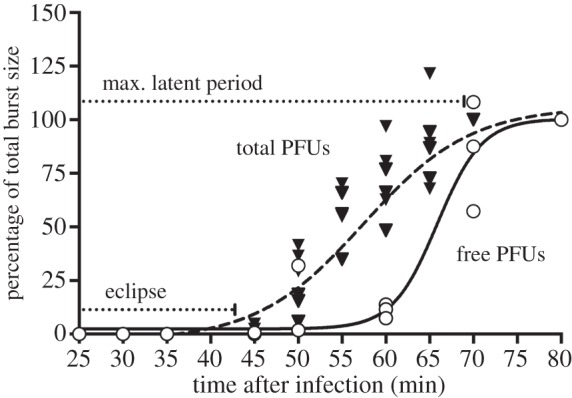
PFU production dynamics of a wt Qß population during infection of *E. coli* cells. Circles represent relative free PFU densities. Triangles represent relative total (free + intracellular) PFU densities. Separate symbols at each time represent different observations. Free and total PFU densities were determined through one-step and intracellular growth assays, respectively, as detailed in §4. The solid and dashed black curves represent sigmoid curves independently fitted to the one-step (*R*^2^ = 0.9395, d.f. = 19) and intracellular (*R*^2^ = 0.8057, d.f. = 51) growth curves, respectively. See text for additional details.

The growth dynamics of a phage during a single cycle of infection may be discerned through two different types of synchronous-growth experiments: intracellular and one-step. In an intracellular growth experiment, the accumulation of total (intracellular + already released) phages, usually as plaque-forming units (PFUs), is determined throughout infection; in a one-step growth experiment, only the amount of free PFUs (in excess of infected but unlysed cells) is determined over time. Preliminary intracellular and one-step growth assays performed with wt Qß (figure 1) showed that, under our experimental conditions: (i) Qß infective progeny (PFUs) become detectable 40–45 min after infection (i.e. the ‘eclipse’ period is 40–45 min long); and (ii) Qß requires ≈70 min (maximum latent period) to complete an infection. Based on these values, we decided to select for an increased *M* through a serial-passage experiment in which synchronously infected *E. coli* cultures would be artificially lysed between 45 and 70 min post-infection. This procedure would enrich a Qß population with rapidly replicating variants. In order to verify that selection was taking place, the PFU yield would have to be monitored in each passage. In addition, the test cultures would have to be processed in parallel with control cultures in which synchronously infected *E. coli* cells would be lysed after the typical Qß maximum latent period of 70 min. Therefore, at *t* = 0, we established six Qß lines: three early-progeny Treatment lines (TA, TB and TC), and three Control lines (CA, CB and CC). In order to (partially) synchronize phage growth during infection, adsorption was confined to an 8-min period. Cell cultures infected with treatment-line phages were artificially lysed at 55 min post-infection (i.e. 55 min after first mixing phages with cells), while in those infected with control-line phages lysis was artificially completed at 80 min post-infection. All lines were serially propagated in fresh *E. coli* cultures for 26 days (see §4 for additional information).

**Figure 2.**
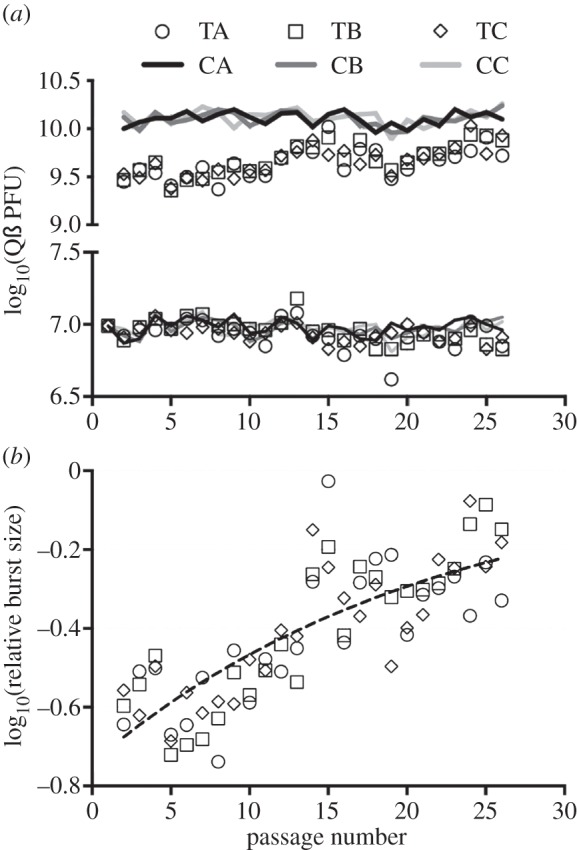
Evolution of Qß PFU production in Treatments (lines TA, TB and TC) and Controls (lines CA, CB and CC) during 26 serial passages. (*a*) PFU yield obtained for each of the lines at each passage. The bottom half of the graph shows the logarithms of the estimated amounts of PFUs added to each experimental culture at the beginning of each passage, while the top half displays the logarithms of the estimated amounts of PFUs produced after the time allowed for infection (55 min for treatments and 80 min for controls). (*b*) Evolution of the amounts of PFUs produced by the treatment lines relative to the corresponding amounts produced by the controls at each passage. Different symbols represent different treatment lines as indicated in figure 2*a*. The dashed curve represents an exponential curve fitted to the data (*R*^2^ = 0.6432, d.f. = 73). See text for additional details.

Figure 2*a* shows the evolution of Qß PFU production in treatments and controls during the selection passages. This figure suggests a small increase in PFU yield in the treatment lines over passages. Indeed, when the data on PFU production collected for each line were independently fitted to a linear trend-line, the slopes of the resulting trend-lines deviated from zero for the three treatments but for none of the controls (data not shown), which indicated that, as figure 2*a* suggests, only in the treatments were the amounts of PFUs produced in each passage significantly increased over the 26 passages. We also determined in each passage the PFU yields of the control lines at 55 min post-infection, and the corresponding data were fitted to linear trend-lines whose slopes showed no significant deviation from zero (data not shown). Moreover, when the PFU yields of the treatments were expressed as percentages of the corresponding average PFUs observed for the controls at every passage, the resultant values fitted an approximately exponential curve (figure 2*b*; *R*^2^ = 0.64, d.f. = 73). Because we started the selection experiment with clonal lines, the relative PFU gains shown in figure 2*b* most probably resulted from selection. Indeed, the exponential overall PFU gain displayed by the treatments is analogous to the trajectory typically observed for the fitness of RNA viruses and other micro-organisms undergoing adaptation to novel environments [12–14]. This trajectory denotes a decreasing fitness gain over time as the populations under selection approach optimality. In RNA viruses, where beneficial mutations may appear in different, competing clones and where the effects of beneficial mutations are usually not independent, this trajectory may also reflect the impacts of clonal interference and epistasis (reviewed in [15]).

### 3.2. Evolution of the multiplication rate and its determinants

The increase in PFU production observed in the treatment lines suggested that their *M* values might have increased. Therefore, fresh lysates of the founder wt population and of the experimental populations obtained at the 25th selective passage were prepared and used to characterize the corresponding populations: WT, obtained from the founder wt Qß population; TA_25_, TB_25_ and TC_25_, each obtained from a different treatment line; and CA_25_, CB_25_ and CC_25_, each obtained from a different control line.

Table 1 shows the *M* estimates obtained for the examined populations. The *M* values of the Treatments_25_ were 1.2- to 1.8-fold larger than that of WT. *M* values were calculated from the corresponding burst size (*B*) and latent period (*L*) means also shown in table 1. *B* and *L* values were determined through sigmoid curve-fitting of a pool of one-step growth curves obtained for each experimental population (see the electronic supplementary material, figures S1–S5), as described in §4. Compared with WT, all Treatments_25_ showed a significantly reduced average *L* (Holm Sidak's Multiple Comparisons Test, adjusted *p*-values as shown in table 1, d.f. = 128), which indicated that the latent periods of the treatment lines decreased during selection. In addition, all Treatments_25_ also exhibited an average *B* 1.2- to 1.7-fold larger than the average *B* of WT. Thus, all Treatments_25_ achieved increased *M* values due to combined increases in their *B* values and decreases in their *L* values. This outcome was unexpected because theoretical and experimental studies on phage latent-period evolution have indicated the existence of a trade-off between latent period and burst size [8,16,17]. Thus, it seems logical that a reduction in the time available for progeny production would reduce the total amount of progeny produced (as long as other phage traits such as eclipse period and maturation rate are kept constant).

**Table 1.** Qß life-history traits estimated for the Treatments_25_, Controls_25_ and WT. Estimates were obtained as detailed in §4.

Qß lines	latent period *L* (min)	burst size *B* (PFU)	multiplication rate *M* (PFUs h^−1^)	maturation rate *m*^a^	adsorption rate *k*^a,b^ (ml min^−1^)
TA_25_	52.86 ± 0.48* (30)	1403	1592	3.27 ± 0.30 (24)	2.01 ± 0.03*** (18)
TB_25_	50.99 ± 0.54***(30)	1746	2054	3.27 ± 0.30 (24)	
TC_25_	52.23 ± 0.46** (28)	2089	2400	3.50 ± 0.48 (24)	
Controls_25_	54.19 ± 0.47 (27)	1614	1787	3.32 ± 0.33 (24)	1.69 ± 0.15 (12)
WT	54.97 ± 0.46 (33)	1213	1324	3.05 ± 0.23 (24)	

*p < 0.01; **p < 0.001; ***p < 0.0001; significance of the difference between each of the Treatments_25_ and WT in *L*, or between Treatments_25_ and WT and Controls_25_ in *k*. See text for further details.

^a^Values represent best-fit or mean values ± s.e.m. Numbers in parentheses represent the numbers of observations used to obtain the estimates; in the case of *k*, both mean estimates—for Treatments_25_ (TA, TB and TC combined) and for WT and Controls_25_ (combined)—were based on a combination of six observations per each experimental population.

^b^Values have been multiplied by 10^9^.

We further investigated the process(es) underlying the changes observed in the parameters *L* and *B*. A reduction in *L* coupled with an increase in *B* suggested an increase in the rates of infection (*sensu stricto*) and/or progeny production. However, the rate at which Qß infects a host cell appeared difficult to measure reliably, and the detailed mechanism of infection remains unknown (reviewed in [18]). Therefore, we did not attempt to determine the infection rates of the experimental populations. On the other hand, and as indicated earlier, phage progeny production involves phage genome expression, replication and progeny maturation. Because of its easy estimation, we used the rate of PFU maturation (*m*) as an indicator of the overall rate of progeny production. Although *m* has been shown to be constant during infection for a variety of phages [19–22], our intracellular growth assays conducted with Qß revealed that the PFU density follows a sigmoid curve characterized by a gradual decrease in the rate of PFU production (or *m*, represented by the slope of the curve), probably due to the termination of the latent period (figure 1). Therefore, a series of intracellular growth curves obtained for each experimental population were fitted to a sigmoid curve (see the electronic supplementary material, figures S1–S5), whose slopes were used as estimates of *m*. The three Treatments_25_ displayed average *m* values higher than for WT (table 1) but the overall differences were not significant (Holm Sidak's Multiple Comparisons Test, adjusted *p*-values > 0.8, d.f. = 100). This result hinted that the expected trade-off between *L* and *B* may have been partly prevented by an increase in *m*.

**Figure 3.**
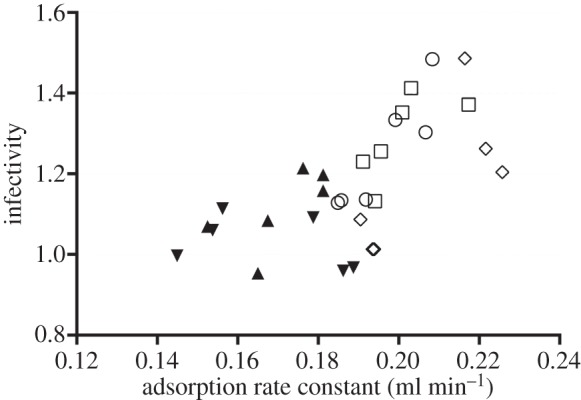
Relationship between infectivity and adsorption in Qß. Different symbols represent observations obtained for different populations: the clear circles, squares and rhomboids stand for TA_25_, TB_25_ and TC_25_, respectively, while the black upright and inverted triangles stand for Controls_25_ and WT, respectively. Adsorption rate constants have been multiplied by 10^8^. Infectivity values represent arcsine transformations of the original percentage values. Correlation analysis between these two parameters indicated, as the figure suggests, a significant positive correlation between them (see text for further details).

Because the reduction in *L* observed for the three Treatments_25_ might also have resulted from an increase in their adsorption rates, we determined the adsorption rate constant (*k*, ml min^−1^) of each of the experimental populations. Altogether, the *k* values estimated for the Treatments_25_ were significantly higher than those obtained for WT and Controls_25_ (table 1; Mann–Whitney test, two-tailed exact *p* < 0.0001). Yet, for this difference to be meaningful, such an increase in *k* should have been paralleled by a gain in infectivity under our typical conditions, where only a minority of the particles has time to adsorb. Phage adsorption to the host cell is commonly described by *N*_t_/*N*_0_ = e^−k*Ct*^, where *C* is the host cell density in the adsorption mixture and *N*_t_/*N*_0_ is the density of phages that remain free after adsorption time *t*. Note that, under these circumstances, *k* only indicates how rapidly free phages disappear from the adsorption mixture but says nothing about the efficiency of adsorption or, more precisely, whether the adsorbed phages actually infect their host cells [23] (although more intricate protocols to determinate phage adsorption rates, based on increases in numbers of infected bacteria over time, could provide this information if needed). Thus, one may enquire whether the increase observed in *k* was accompanied by a parallel increase in infectivity. We investigated whether such a relation existed through correlation analysis between the infectivity values (determined as detailed in §4) and the *k* values estimated for all of the experimental populations (figure 3). Results from such analysis (Pearson's *r* = 0.6468, *R*^2^ = 0.4183, two-tailed *p* = 0.0001) indicate that, as figure 3 suggests, in Qß, infectivity under our conditions is proportional to *k* and that the increase in *k* observed for the Treatments_25_ was paralleled by an increase in infectivity.

Overall, the degree of phenotypic evolution varied among the experimental populations. Owing to the randomness of mutagenesis, the occurrence of phenotypic differences among parallel populations in evolution experiments is expected. Here, however, some phenotypic variability might have also resulted from small differences in the manipulation of the parallel experimental lines during the selection experiment (see §4 for details). Thus, TC_25_ displayed the greatest values of *M*, *B* and *m*, while TB_25_ showed the smallest average *L*. Controls_25_ also showed some degree of phenotypic evolution, probably as a result of an adaptation to grow in *E. coli* NR16205 cells under our experimental conditions. It is worth noting here that, during the serial-passage experiment, artificially lysing the experimental cell cultures infected with control lines 80 min after infection might have terminated longer latent periods, hence selecting for phage variants with a progeny production higher than that of wt Qß, as in the case of the treatment lines. Thus, the phenotypic evolution observed for the Controls_25_ was in the same direction as the evolution observed for the Treatments_25_, namely increased *M*, *B*, *m* and *k* and decreased *L*, but all these changes were subtle and, at least for *L* and *m*, which could be statistically analysed, did not render values significantly different from those observed for WT.

### 3.3. Evolution of Qß plaque-forming unit virion stability

**Figure 4.**
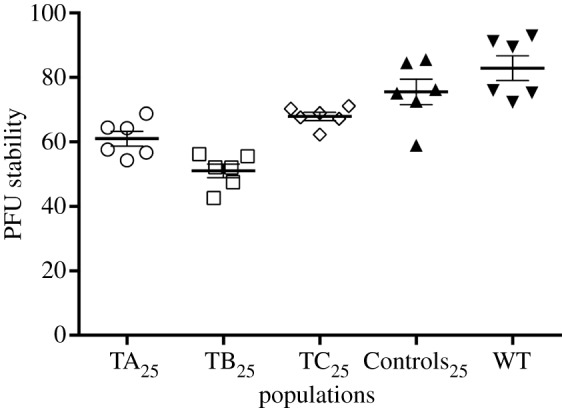
PFU stability (expressed as percentage) of the wt and evolved Qß populations. Bars represent means ± s.e.m. (*n* = 6).

PFU stability, which represents the virion's capability to remain infectious over time, was estimated from the residuum of free PFUs in a phage suspension after 6 h of incubation in LB medium at 37°C with gentle shaking. An endpoint measure was preferred over a rate because the evolved populations were anticipated to be genetically heterogeneous and their PFU decay rates were thus expected to vary over time and to be difficult to compare, as previously observed with phage T7 [24]. The results showed that the three Treatments_25_ have low PFU stability compared with WT (figure 4). When the stability values of the Treatments_25_ were combined and compared with the corresponding values of WT and Controls_25_ (also combined), the difference was significant (Mann–Whitney test, two-tailed exact *p* < 0.0001).

These results, together with the changes observed in *M*, imply a trade-off between Qß fecundity and lifespan. What may be the mechanism(s) underlying this trade-off? In general, PFU decay may be due to the degradation of any of the virus components and consequent loss of virion integrity. For instance, in the ssRNA virus causing foot-and-mouth disease, genome insertions decreased virion thermal stability [25], while large deletions (greater than 400 bases) increased virion stability and lifespan [26]. Similarly, across a wide range of double-stranded DNA (dsDNA) coliphages, PFU decay rate was positively correlated with the density of the packaged genome, although there was no correlation between this trait and the multiplication rate [6]. Among the phage traits analysed, only the capsid surface mass (the molecular weight of the capsid shell divided by the surface of the capsid) was negatively correlated with both mortality and multiplication rates, suggesting that the lesser the relative amounts of time and energy invested in the production of each phage particle, the higher the mortality rate [6].

**Figure 5.**
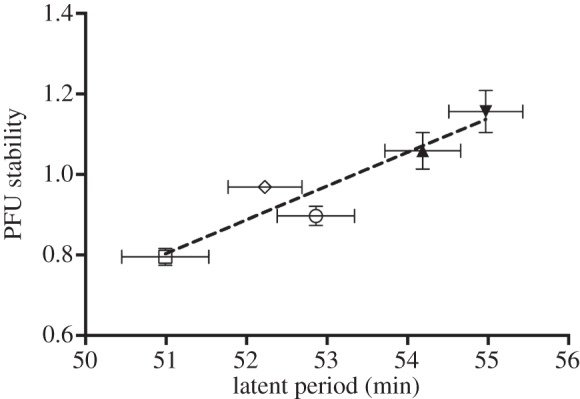
Relationship between PFU stability and latent period in Qß. Different symbols represent PFU stability and latent period means obtained for different populations: the clear circles, squares and rhomboids stand for TA_25_, TB_25_ and TC_25_, respectively, while the black upright and inverted triangles stand for Controls_25_ and WT, respectively. For each symbol, bars stand for the s.e.m. of the corresponding parameter (PFU stability and latent period). The numbers of observations per population are the same as those indicated in table 1 and figure 4 for latent period and PFU stability, respectively. PFU stability values represent arcsine transformations of the original percentage values. The dashed black curve represents the nonlinear regression of PFU stability on latent period (nonlinear lines regression, *R*^2^ = 0.6463, d.f. = 28).

Although our data do not lend themselves to statistical inferences on the possible mechanistic causes for the fecundity/lifespan trade-off reported here for Qß, it is notable that the experimental population showing the lowest PFU stability values, TB_25_, was the one showing the shortest latent period. Although the experimental population displaying the highest average *B* was TC_25_, the difference in average *B* between TB_25_ and TC_25_ is probably not significant considering the variability in burst size usually observed for Qß [27]. Overall, these results seem to suggest that the less the amount of time that Qß needs to invest in progeny production (due, for instance, to a faster but less stable folding of the progeny genome), the shorter is the lifespan of the progeny. Likewise, as figure 5 shows, PFU stability and latent period showed a strong positive relationship among the experimental populations (nonlinear lines regression, *R*^2^ = 0.6463, d.f. = 28).

### 3.4. Evolution of Qß growth rate

**Figure 6.**
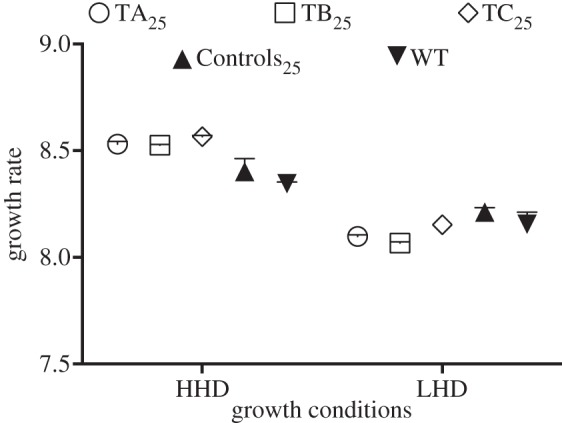
Growth rates of the wt and evolved Qß populations under conditions of high and low host-cell density (HHD and LHD, respectively). Growth rates are in PFU doublings per hour. Symbols represent medians and ranges (*n* = 3).

Empirical evidence of a trade-off between virion fecundity and stability in a challenging environment was recently shown for the dsDNA coliphage T7 [24], wherein T7 virions adapted to survive in 6 M urea displayed a reduced growth rate in cell culture. Similarly, a trade-off between virion survival and reproduction was analysed for the dsRNA phage φ6 in another recent study [28], wherein phage populations adapted to periodic heat shocks displayed decreased fitness. A confounding aspect of these two reports is that, contrary to multiplication rate, both phage growth rate and fitness involve survival *per se*, and hence their estimation depends strongly on local environmental conditions. For example, all other things being equal, increased virion stability might or might not be coupled to decreased growth rate or fitness depending on the host-cell density: if this density is low enough to render a competition advantage to those virions showing higher stability, increased stability would be coupled to an apparent increased growth rate or fitness. To illustrate this example, figure 6 summarizes the results of a set of assays in which we determined the growth rates of our experimental populations under conditions of low and high host-cell density (LHD and HHD, respectively). Under LHD conditions, Treatments_25_ showed significantly lower growth rates than WT and Controls_25_ (Mann–Whitney test, two-tailed exact *p* = 0.0110). This result suggests that, under these conditions, the increased *M* values displayed by the Treatments_25_ were insufficient to compensate for their decreased PFU stabilities between consecutive infections. Conversely, under HHD conditions, in which the dispersal period (the time between two consecutive infections) was reduced to a minimum, Treatments_25_ showed growth rates significantly higher than those of WT and Controls_25_ (Mann–Whitney test, two-tailed exact *p* = 0.0004), in agreement with their higher *M* values. These results show that, when a trade-off between fecundity and any other component of fitness is analysed, special care must be taken in the parameter used to determine changes in the reproduction ability.

When the constraints that govern phage life-history evolution are analysed within the framework of the optimal foraging theory (concerning the adaptive evolution of animal foraging behaviour), the latent period equals the residence period, i.e. the time spent feeding on a particular source of energy (in this analogy, a bacterial cell; reviewed in [29]). According to the marginal value theorem of the optimal foraging theory, when energy sources are found in patches, the residence period is expected to evolve towards an optimum that ensures the highest fitness available [30]. When applied to phage evolutionary biology, this theorem predicts that phages would evolve shorter latent periods in environments containing high densities of susceptible host cells [17]. This prediction has been empirically supported in a number of experiments [8,9], although attempts to reach an optimal latent period have failed, probably because of unidentified constraints [10]. Our results also seem to endorse such a prediction because the evolution of shorter Qß latent periods produced higher growth rates under HHD conditions, and vice-versa.

### 3.5. Genetic evolution

The Qß genome contains 4217 bases organized in three cistrons that encode a total of four proteins whose functions are well determined: (from 5′ to 3′) A2, which mediates both adsorption to the host cell and post-replicative host lysis; the Coat protein and its elongated A1 or Read-Through version, which is required for progeny maturation; and the catalytic ß subunit of the Qß replicase. With the aim of determining which specific changes in the genes that code for these proteins produced the observed phenotypic changes, the consensus genome sequences of WT and of the populations resulting from the 25th and 26th selective passages were obtained and analysed. The genetic changes, all of them polymorphisms, detected for each of the Treatments_25_ and Controls_25_ relative to the founder wt population, are shown in table 2. The same polymorphisms (with slight modifications in the corresponding percentages) were observed for both the Treatments_26_ and the Controls_26_ (data not shown).

**Table 2.** Genetic changes in the consensus genome sequence of each of the Treatment_25_ and Control_25_ populations relative to the ancestral wt Qß population. Percentages represent the proportion of the observed change. The function of the proteins encoded by the genes A2, A1 and ß subunit is adsorption and lysis, virion assembly and genome replication, respectively. UTR, untranslated region.

site	change	protein	percentage changes					
			TA	TB	TC	CA	CB	CC
47	G → U	5′-UTR	5–25					
	G → A		5–25					
52	A → G	5′-UTR	5–25		5–25			
143	U → C (L28P)	A2	50–75	>75	>75	25–50	25–50	25–50
149	U → C (I30T)	A2				25–50	25–50	25–50
1294	U → C (F412L)	A2	5–25					
1649	C → U (T102T)	Coat/A1		5–25				
1915	A → G (N191S)	A1	5–25					
1930	A → G (Q196R)	A1	5–25		5–25			
2001	C → U (L220F)	A1	5–25	25–50	5–25			
2016	U → C (F225L)	A1	25–50	25–50	25–50			
	U → G (F225V)		25–50	25–50	25–50	5–25	5–25	25–50
3945	G → A (G532S)	ß subunit			25–50			

A total of 11 single-base-substitution polymorphisms were detected: two in the 5′ untranslated region (5′-UTR), three in the A2-coding gene, five in the A1-coding gene and one in the gene encoding the ß subunit of the Qß replicase. Two of the polymorphisms (at sites 47 and 2016) exhibited two different non-wt alleles each. In the 5′-UTR, one of the two detected polymorphisms was present only in the TA lineage while the other was also present in TC (table 2). These two polymorphisms mapped at genome positions 47 and 52. Both positions are located in a sequence of the Qß genome (bases 47–57) that forms a hypothetical long-distance structural interaction with another sequence of the Qß genome (bases 459–474) [31]. In addition, position 52 maps in the binding-site sequence (bases 50–78) of the ribosomes during A2-translation initiation [32]. Thus, we may reasonably venture that the 5′-UTR polymorphisms affect Qß genome folding and/or translation. Interestingly, changes at the same two positions of the Qß 5′-UTR have previously been observed in Qß populations evolved in *E. coli* cultures [33], which further suggests that these positions are involved in one or more important functions of the Qß life cycle.

In the gene that encodes the A2 protein, we detected three polymorphisms, located at genome positions 143, 149 and 1294 (table 2). The first two positions map in the amino-terminal half of A2, where at least the lytic function of this protein resides [34]. The polymorphism detected at position 143 is the only one present in all treatments and controls. This polymorphism comprises a U → C mutation that reached a substantially higher frequency in the treatments than in the controls and was almost fixed in TC. These observations, combined with the phenotypic results, suggest that this change is related to the increased adsorption rate constant (*k*) observed for Treatments_25_ and Controls_25_. Thus, there is some parallelism across the experimental populations between the frequency shown by this particular mutation and the changes observed in *k*. This mutation, which translates as L28P in A2, was previously observed among Qß A2 mutants able to lyse *E. coli* cells resistant to the lytic function of wt A2 [34], which indicates that it may modify the lytic activity of A2. Our results further suggest that this mutation may also modify the attachment activity of A2 and thus that both functions of A2 (lysis and attachment) may map to close regions of A2. The polymorphism detected at position 149 of the A2-coding gene, which also comprised a U → C mutation, was present in all controls but in none of the treatments (table 2). During the selection experiment, only the control lines were given sufficient time in each passage to lyse their host cells, so that this specific change might be related to the lysis function of A2.

Interestingly, among treatments, the gene encoding the Coat/A1 protein accumulated the greatest number of detectable polymorphisms, mostly in the part of the gene exclusively involved in coding A1. (A1 is translated when a ribosome incorporates tryptophan at the natural UGA stop codon of the Coat-coding gene located at position 1743 of the Qß genome.) The changes observed in the A1-coding gene might be linked to the increase in the rate of PFU maturation (*m*) observed for all the treatments if this increase were actually due to an enhanced efficiency for Qß assembly, which, unfortunately, we cannot verify with the available data. All the non-synonymous polymorphisms detected in the A1-coding gene were located in a sequence of about 100 bases (genomic positions 1915–2016) of the total 993 bases coding for A1, which indicates that residues important for the function of A1 map in this region. One additional polymorphism (at position 1649), observed only in TB, involved a synonymous change, suggesting that this polymorphism may serve a structural function [35].

Finally, we detected a polymorphism in the ß-subunit gene that maps in the bridge domain of the Qß replicase [36]. This domain is suggested to interact with the Qß replicase cofactors EF-Tu and S1 and is probably also involved in unwinding the duplex between the template and the nascent portion of the product when the duplex reaches a maximum size of six to seven base pairs [36]. Thus, the observed change in the ß subunit may have modified Qß RNA synthesis initiation and/or extension. Interestingly, this change was observed only in TC, which also displayed the highest overall multiplication rate.

All the analysed populations showed genetic evolution, although the 10 polymorphisms that the treatments accumulated exceeded the three in the controls. Among the treatments, TA acquired the most polymorphisms (eight), followed by TC (with six) and TB (with four). In these populations, convergent evolution (the same polymorphisms showing similar frequencies) was observed at three genome positions (143, 2001 and 2016), and two other convergent changes were observed between TA and TB (at genome positions 52 and 1930). Among the controls, the same three changes were detected in the three populations, two of these polymorphisms also being observed among the treatments (table 2).

Convergent evolution is a common result of evolution experiments carried out with RNA viruses (reviewed in [37]). Apart from the obvious reason that, in this type of experiment, parallel virus lines are challenged with a set of identical environmental conditions, there are some unique characteristics of RNA viruses that favour convergent evolution. Notable among these are the shortness, compactness and structural constraint of their genomes, which offer a limited array of adaptive responses to a given environmental change [38]. In addition, antagonistic epistasis and clonal interference, which are thought to be common in RNA virus populations (reviewed in [15]), would allow the same beneficial mutations with strong effects to be preferentially fixed in most, if not all, parallel evolving populations.

### 3.6. Concluding remarks

The present report documents a fecundity/lifespan trade-off for the ssRNA coliphage Qß. This trade-off was predicted to exist in coliphages and implies constraints in the evolution of phage life history similar to those observed in higher eukaryotes. However, conclusions must be made cautiously because results from a short-term evolution experiment similar to that described here may differ from those obtained in long-term evolution experiments. Further investigation might disclose whether the observed trade-off could be lessened (or perhaps even eliminated) via additional molecular changes resulting from mutations that compensate for the lifespan deficit.

An interesting outcome of evolution experiments carried out with viruses continuing to adapt to their usual host or adapting to a new one is that the genetic changes resulting from that adaptation may inform about the genes, sequences and/or specific sites where important virulence factors map [39]. In addition, analysis of such genetic changes may provide information on negative genetic correlations between different viral life-history traits. In the long term, a better overall understanding of virus evolution and its constraints may help to design better vaccines, diminish the virulence of present pathogens, or even anticipate the emergence of highly virulent strains in the future.

## 4. Material and methods

### 4.1. Phages, bacteria, growth media and general procedures

Wild-type Qß was obtained by expressing the plasmid pQßm100 [40], which was the gift of Donald R. Mills (SUNY Downstate Medical Center, Brooklyn, NY, USA). Qß phages were always propagated in exponentially growing cultures of *E. coli* strain NR16205 (which is a *ΔyeeD*::*tet* derivative of NR10836 *ara thi* *Δ*(*pro-lac*) F′CC106 [41]), provided by Roel M. Schaaper (National Institute of Environmental Health Sciences, Research Triangle Park, NC, USA).

Cell transformations with pQm100 were performed using CaCl_2_ [42]. Unless otherwise indicated, cells were grown in Luria-Bertani medium (LB) supplemented with 2 mM CaCl_2_ and 15 µg ml^−1^ tetracycline (Tet). Phages were plated using 30 ml of LB bottom agar with 2.0 per cent Bacto agar by mixing 0.1 ml of a phage suspension with 0.25 ml of a cell culture at optical density 600 nm (OD_600_) ≈ 0.5, which, under our experimental conditions, is a cell density of ≈10^8^ colony-forming units per ml (CFU ml^−1^). For all platings, the top agar (3 ml) contained 0.4 per cent Sigma-Aldrich Noble agar, except for those carried out during the one-step growth experiments where it contained 0.8 per cent Bacto agar. The top agar was always prepared in distilled water. Inoculated plates were incubated for about 12 h at 37°C.

When needed, synchronized phage growth was achieved by restricting the time for adsorption to 8 min at 37°C with gentle shaking, after which infected cultures were centrifuged for 1 min at 8000*g* in a bench centrifuge at room temperature and washed twice to remove unadsorbed phages.

Plate lysates were prepared as previously described [27].

### 4.2. Selection for increased Qß multiplication rate

The selection experiment was started with six separate Qß experimental lines: three Treatments (lines TA, TB and TC) and three Controls (lines CA, CB and CC). All these lines were independently established at *t* = 0 by mixing ≈10^7^ wt Qß PFUs with ≈10^8^ host CFUs. Mixtures were incubated at 37°C with gentle shaking for 8 min to allow phage adsorption. Cells were then pelleted, washed twice and re-suspended in 5 ml LB (not supplemented with CaCl_2_), and infection was allowed to proceed at 37°C for 55 min (treatments) or 80 min (controls). At each endpoint, infection was stopped by adding 125 µl of chloroform to the experimental cultures. Chloroform ruptures the bacterial cell wall, thus allowing the release of the cell contents; in the case of infected cells, this affects the release of accumulated progeny phage. The resulting lysates were titred and appropriate dilutions of each were prepared in order to infect a new cell culture following the same above steps. This protocol was applied to each experimental line for 26 passages. At each passage, treatments sets and controls sets were manipulated separately, while the three lines in each of these two sets were manipulated in parallel. Because of this *modus operandi*, small differences (no more than 30 s) in adsorption and total infection times among treatments or control lines were inevitable and might have influenced, among other factors, the occurrence of genetic (and phenotypic) differences among parallel cultures during evolution.

Samples of the founder wt Qß lysate and of each lysate generated during the selection experiment were mixed with glycerol (1 : 1 vol/vol) and stored frozen at −20°C. Once the selection experiment was completed, samples from the frozen lysates of the founder population and of the populations obtained after the 25th and 26th passages were used to produce fresh lysates that were then used to characterize the wt and the 25th-passage populations, and for sequencing, as detailed below.

### 4.3. One-step and intracellular growth curves

One-step growth curves were obtained as previously described [27]. In brief, 0.1 ml of a phage suspension was mixed with 0.9 ml of a cell culture (≈10^8^ CFU ml^−1^) at a multiplicity of infection (MOI) ≈ 0.01 and adsorption was allowed to proceed as detailed earlier. Cells were then pelleted, washed twice and serially diluted in LB (not supplemented with CaCl_2_). The 10^3^- and 10^5^-fold dilutions of the originally infected culture were held at 37°C with gentle shaking for 1.5 h, and 100-µl samples were retrieved from one or another dilution every 5 min and plated with NR16205 cells. Plates were incubated overnight and the resulting titres were used to estimate free PFU densities over time.

PFU production dynamics during a single-infection cycle were studied using essentially the same procedure as above except that the retrieved samples were mixed with 0.9 ml D broth (0.2% Bacto tryptone, 0.5% NaCl) containing 25 µl of chloroform before plating with NR16205 cells.

Three growth assays of each type were conducted in parallel for each experimental population except for the Controls_25_, for which one experimental culture was assayed for each population. The resulting one-step and intracellular growth curves (see the electronic supplementary material, figures S1–S5) were fitted using nonlinear regression to a sigmoid curve defined as 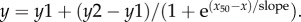 In fitted one-step growth curves, the estimated (best-fit) values of *y*1 and *y*2, which represent the initial (or infective) and final (or produced) PFUs, respectively, were used to determine the burst size of each experimental population as 10^(*y*2−*y*1)^. In the same curves, the values obtained for *x*_50_, which represents the time at which half of the progeny has been released from the infected cells, were used as estimates of the latent period. In the case of the intracellular growth curves, the values obtained for the slope were used as estimates of the PFU maturation rate.

### 4.4. Adsorption rate and infectivity estimates

For the adsorption assays, 0.1 ml of a phage suspension was mixed with 0.9 ml of a cell culture (≈10^8^ CFU ml^−1^) at a MOI ≈ 0.01, and adsorption was allowed to proceed as previously. Cells were then pelleted and washed twice and the supernatants were carefully collected and mixed with 0.1 ml of chloroform. Three samples were retrieved from each assay: one from the original phage suspension to determine the total amount of PFU added to the experimental cell culture (*P*_T_); one from the supernatants to determine the total amount of unadsorbed PFU (*P*_U_); and one from the resuspended pellet to determine the number of adsorbed PFU able to produce progeny PFU. The adsorption rate constant (*k*, ml min^−1^) was estimated from: *P*_U_ = *P*_T_e^−k*tC*^, where *t* is the time for adsorption and *C* is the density of CFU in the adsorption mixture. Infectivity was estimated as the percentage of *P*_T_ that produced plaques.

Two different sets of experiments, each comprising three parallel experimental cultures, were conducted with each of the experimental populations, except for the Controls_25_, for which a total of two independent experimental cultures were assayed for each population.

### 4.5. Plaque-forming unit stability estimates

PFU stability assays were started by adding 10 µl of a phage suspension (≈2 × 10^7^ PFU ml^−1^) to 1 ml of LB and incubating at 37°C with gentle shaking. After 6 h, dilutions of each phage suspension were plated with NR16205 cells to determine the corresponding PFU titres. The number of different experimental cultures assayed per population was as detailed for the adsorption and infectivity assays. PFU stability was then estimated for each experimental population as the percentage of initial PFUs that were able to plaque at the end of the experiment.

### 4.6. Growth rate estimates

Two different sets of growth experiments were conducted: one at high host-cell density (HHD), the other at low host-cell density (LHD).

In the HHD assays, 0.1 ml of a phage suspension (≈10^7^ PFU ml^−1^) was added to 5 ml of a cell culture (≈10^8^ CFU ml^−1^) and the resulting mixture was incubated at 37°C with gentle shaking. After 1.5-h incubation, when at least one infection cycle had been completed and cell cultures had reached the end of the log phase, a 0.1-ml sample was retrieved from each culture and independently mixed with 0.9 ml of D-broth containing 25 µl of chloroform. A 0.1-ml sample from this mixture was then used to infect the next experimental cell culture. After three consecutive passages, the titre of the final experimental culture was determined and growth rate was estimated as log_2_(*P*_t_/*P*_0_)/*t* (PFU doublings per hour), where *P*_t_ is the number of PFU produced after 4.5 h corrected for dilutions over the multiple passages. (Note that this growth rate estimator, which indicates the number of times a phage population doubles per hour, does not at all represent the molecular mode by which Qß reproduces and is used only for purposes of comparison.)

In the LHD growth, 0.1 ml of a phage suspension (≈10^6^ PFU ml^−1^) was mixed with 0.9 ml of a cell culture (≈10^8^ CFU ml^−1^) and adsorption was allowed to proceed as usual. Cells were then pelleted, washed twice and diluted 10^3^-fold in LB (not supplemented with CaCl_2_). The resulting mixture was incubated at 37°C with gentle shaking for 3 h. A 0.1-ml sample was then retrieved and mixed with 0.9 ml of D-broth containing 25 µl of chloroform, and adequate dilutions of this mixture were prepared and plated with NR16205 cells to determine the corresponding titres. Growth rates were estimated as indicated before.

Three growth assays of each type were conducted in parallel for each experimental population, except for the Controls_25_, for which one experimental culture was assayed for each population.

### 4.7. Sequencing

General procedures for phage RNA purification and sequencing were as described previously [27]. Additionally, and in order to sequence the 5′ and 3′ ends, RNA samples were also handled as detailed in [33]. The primers used in the RT, PCR and sequencing reactions, and the PCR cycling parameters, are summarized in the electronic supplementary material, table S1. The entire consensus genome sequences (except for the first 40 bases of the 5′ untranslated region) of the wt and of the 25th- and 26th-passage populations were determined. Sequence files were analysed with Lasergene's SeqMan Pro software (v. 8.0.2; DNAStar, Inc., Madison, WI, USA).

### 4.8. Statistical analyses

All statistical analyses were conducted with GraphPad Prism (v. 6.0a for Mac OS X for Macintosh, GraphPad Software, La Jolla, CA, USA, www.graphpad.com) according to Sokal & Rohlf [43].

## Supplementary Material

García-Villada & Drake_ESM

## Data accessibility

All the data that support the results shown and discussed in the present paper are available under request at garciavillal@niehs.nih.gov.
