# Supplementary material for: Experimental selection reveals a trade-off between fecundity and lifespan in the coliphage Qß
Source: Open Biol. 2013 Jun;3(6):130043. doi: 10.1098/rsob.130043 (PMC3718328; doi:10.1098/rsob.130043)

**Table and Figure Captions to ESM**

**Table S1.** Primers and RT, PCR, and sequencing parameters used for RT, amplifying, and sequencing.

**Table S1 footnote.** PCR reactions were carried out with PfuTurbo DNA polymerase (Stratagene). Amplification products were prepared for sequencing using BigDye Terminator v3.1 (Applied Biosystems).

^a^The remaining parameters used in the PCR and sequencing reactions were as specified by the PfuTurbo DNA polymerase and BigDye Terminator manufacturers’ instructions, respectively. In all cases, the second segment of the PCR was cycled 35 times.

**Figures S1–S5.** Growth curves of TA_25_, TB_25_, TC_25_, Controls_25_, and WT, respectively. For each figure, (a) and (b) represent a series of one-step and intracellular growth curves, respectively, for each experimental population. At each time point, symbols are staggered for clarification. In the intracellular growth curves, PFU densities at 30 and 35 min post-infection represent the amounts of infecting phages in the experimental cultures; because preliminary experiments indicated that none of the Qß experimental populations was able to produce infective progeny before 35 min after infection, samples retrieved at those two times were directly plated rather than mixed with chloroform. The dashed curves represent the sigmoid curves fitted to each of the growth curves through non-linear regression analysis. The goodness-of-fit of each of these are shown in parenthesis in each corresponding graph.

**Table S1.** Primers and RT, PCR, and sequencing parameters used for RT, amplifying, and sequencing.

| **reactions** | **primer name** | | **primer sequence (5′→3′)** | **reaction conditions** | **product name** |
| --- | --- | --- | --- | --- | --- |
| **RT** |  | |  |  |  |
| Qß genomic RNA | Rev_1 | | GATCCCCCTCTCACTCGT | 37ºC,  2 h | ß-subunit F |
|  | Rev_2 | | TCGTGCCCTGGAAGACC |  | ß-subunit S |
|  | Rev_3 | | acttctcccaggcaacagctt |  | RT |
|  | Rev_4 | | TCGGTACTATACTGCGTGAAC |  | A2 F |
|  | Rev_5 | | ATGATATAGCTGACCTTGTTGAG |  | A2 S |
|  | Rev_6a | | CCAGTGAGCAGAGTGACGAGGACTCGAGCTCAAGCTTTTTTTTTTTTTTTTT |  | 3′ end |
| **PCR^a^** |  | |  |  |  |
| ß-subunit  F | Rev_1 | | GATCCCCCTCTCACTCGT | Annealed  at 62ºC | ß-subunit F2 |
|  | For_1 | | GCGGCAAGCACTACTATTCT |  |  |
| ß-subunit  S | Rev_2 | | TCGTGCCCTGGAAGACC | Annealed  at 65ºC | ß-subunit S2 |
|  | For_2 | | CCGATGGCGTGATAGTTG |  |  |
| RT | Rev_3 | | acttctcccaggcaacagctt | Annealed  at 68ºC | RT2 |
|  | For_3 | | GCAATCTTCCGTTCGCTACAC |  |  |
| A2 F | Rev_4 | | TCGGTACTATACTGCGTGAAC | Annealed  at 66ºC | A2 F2 |
|  | For_4 | | aatatctcgggcttttactgc |  |  |
| A2 S | Rev_5 | | ATGATATAGCTGACCTTGTTGAG | Annealed  at 63ºC | A2 S2 |
|  | For_5 | | ttcactgagtataagaggaca |  |  |
| A2 S | Rev_7 | | CGGTAACCCTCACGCAGTCT | Annealed  at 48ºC | 5′ end |
|  | Rev_6a | | CCAGTGAGCAGAGTGACGAGGACTCGAGCTCAAGCTTTTTTTTTTTTTTTTT |  |  |
| 3′ end | Rev_6b | | ACGAGGACTCGAGCTCAAGC | Annealed  at 68ºC | 3′ end2 |
|  | For_6 | | ACCTCTTCTCGCGTTGTCTCT |  |  |
| **sequencing** | |  |  |  |  |
| ß-subunit  F2 | For_6 | | ACCTCTTCTCGCGTTGTCTCT | Annealed  at 50ºC | ß-subunit F2_F1 |
| ß-subunit  S2 | For_7 | | GTGCCATACCGTTTGACTTC |  | ß-subunit F2_F1 |
|  | For_8 | | CTCGGGCTTTGAAGTATGTTTTAG |  | ß-subunit F2_F2 |
|  | For_9 | | GGAAGTTTTTAAGTATGTTGGTTT |  | ß-subunit F2_F3 |
|  | Rev_8 | | CCGTAGGGTGCCAGAAC |  | ß-subunit S2_R1 |
|  | Rev_9 | | AAACCAACATACTTAAAAACTTCC |  | ß-subunit S2_R2 |
|  | Rev_10 | | CTGATATCGAAATGTGTAGAAGC |  | ß-subunit S2_R3 |
| RT2 | For_10 | | ATTAACCCAACGCGTAAAGC |  | RT2_F1 |
|  | For_11 | | CGCAGTATAGTACCGATGAGG |  | RT2_F2 |
|  | For_12 | | CGGCTTAGTTATACCACGTTC |  | RT2_F3 |
|  | Rev_11 | | GAGAGAGTTACGCGAAGATGC |  | RT2_R1 |
|  | Rev_12 | | GTCGCATCAAGGTCAATATAA |  | RT2_R2 |
|  | Rev_4 | | TCGGTACTATACTGCGTGAAC |  | RT2_R3 |
| A2 F2 | For_4 | | aatatctcgggcttttactgc |  | A2 F2_F1 |
|  | For_13 | | cagcttcgttgttgattggttc |  | A2 F2_F2 |
|  | Rev_4 | | TCGGTACTATACTGCGTGAAC |  | A2 F2_R1 |
|  | Rev_5 | | ATGATATAGCTGACCTTGTTGAG |  | A2 F2_R2 |
| A2 S2 | For_14 | | cggagccgataatgaaaT |  | A2 S2_F1 |
|  | For _4 | | aatatctcgggcttttactgc |  | A2 S2_F2 |
|  | Rev_7 | | CGGTAACCCTCACGCAGTCT |  | A2 S2_R1 |
|  | Rev_5 | | ATGATATAGCTGACCTTGTTGAG |  | A2 S2_R2 |
| 5′ end | Rev_6b | | ACGAGGACTCGAGCTCAAGC |  | 5′ end_F |
|  | Rev_7 | | CGGTAACCCTCACGCAGTCT |  | 5′ end_R |
| 3′ end2 | For_6 | | ACCTCTTCTCGCGTTGTCTCT |  | 3′ end2_F |
|  | Rev_6b | | ACGAGGACTCGAGCTCAAGC |  | 3′ end2_R |

**Figure S1**


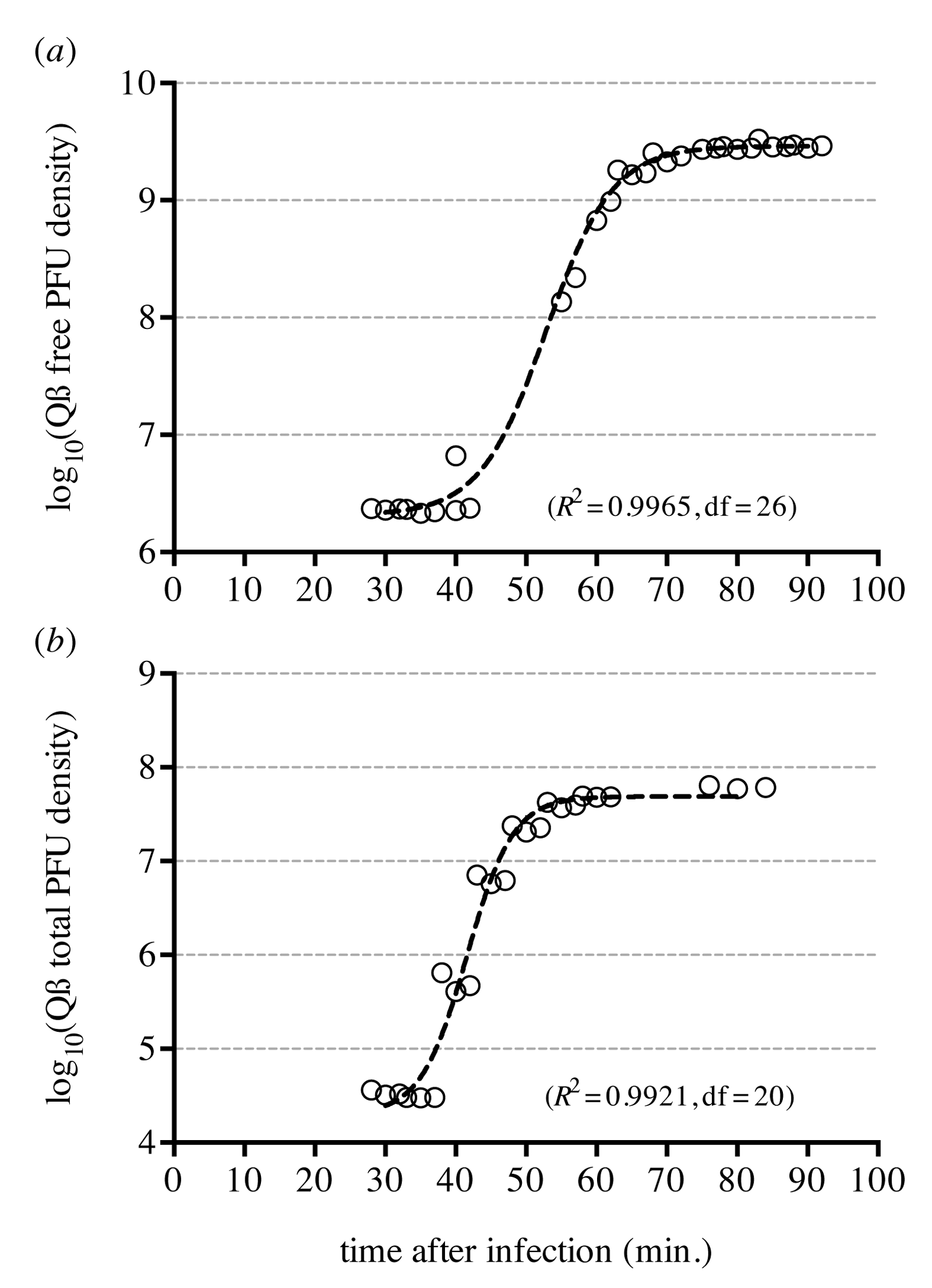


**Figure S2**


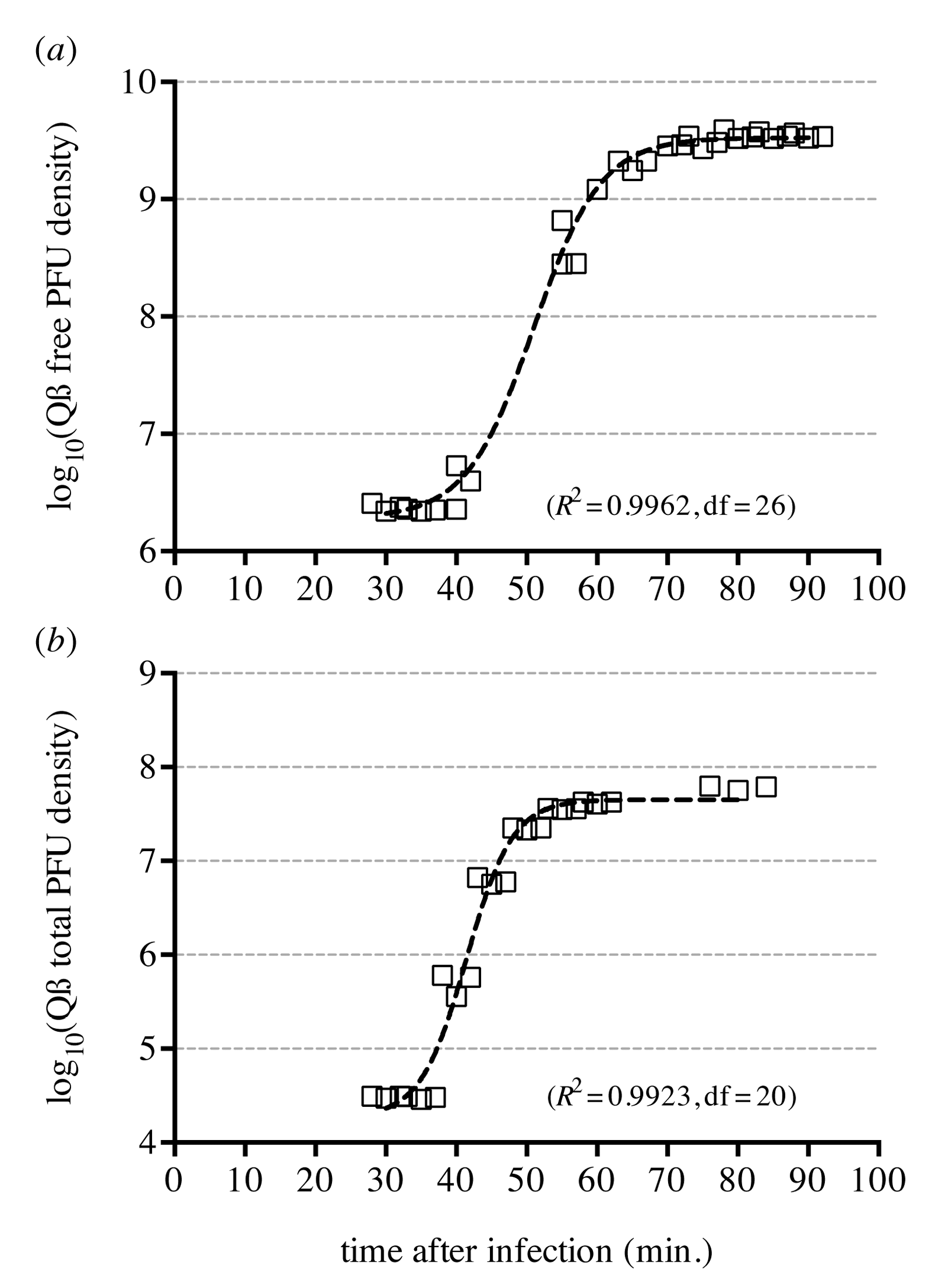


**Figure S3**


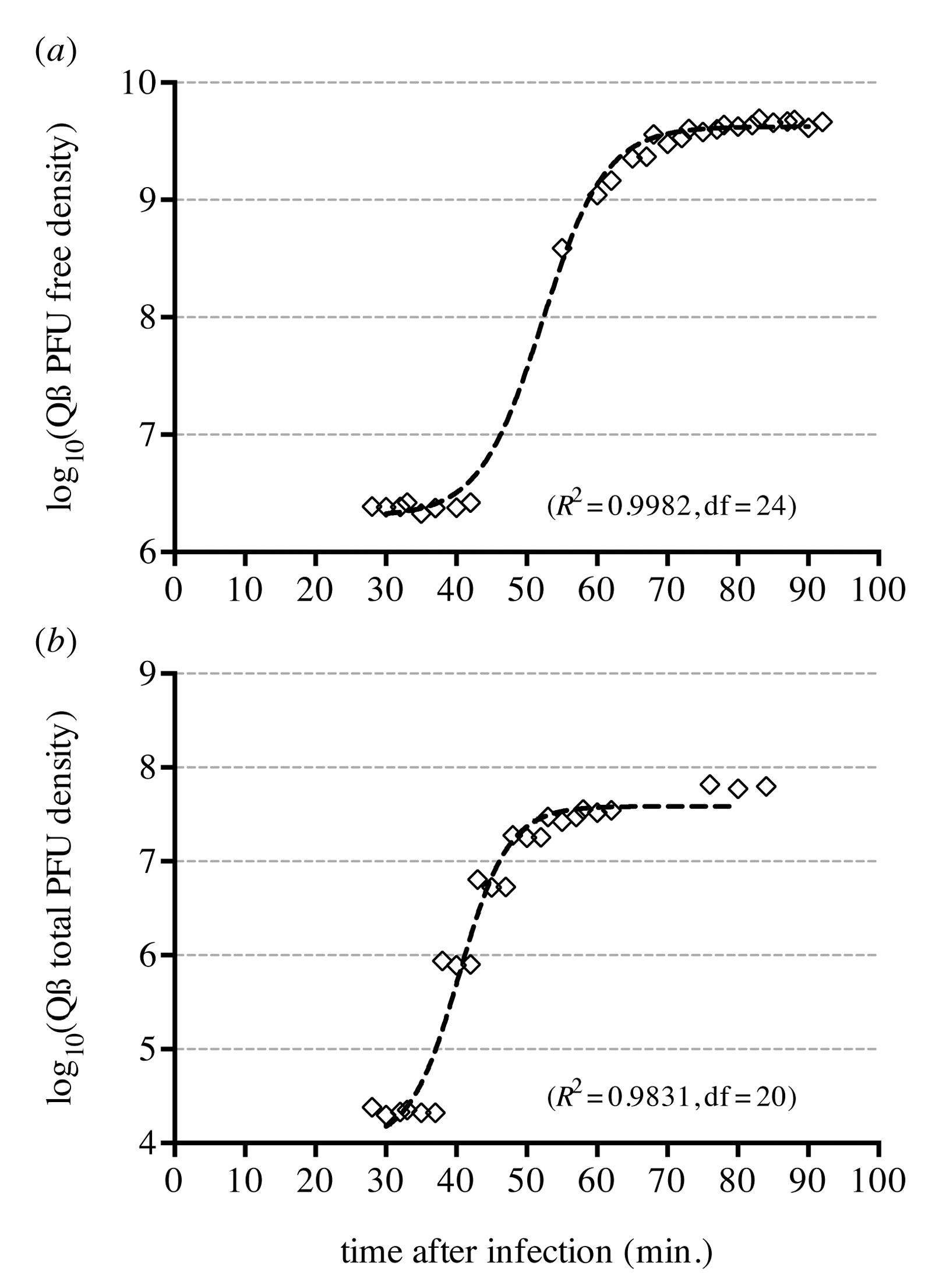


**Figure S4**


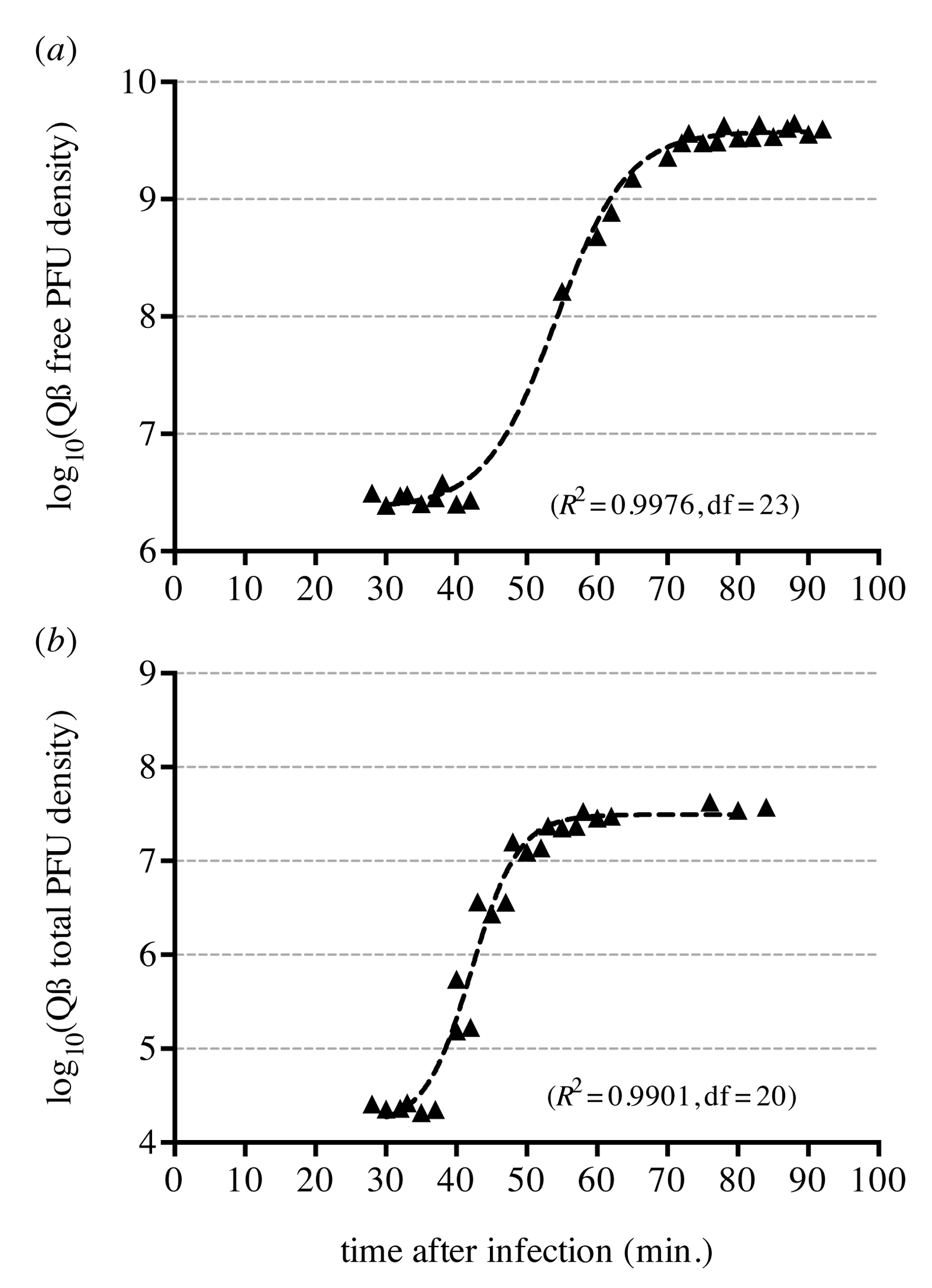


**Figure S5**


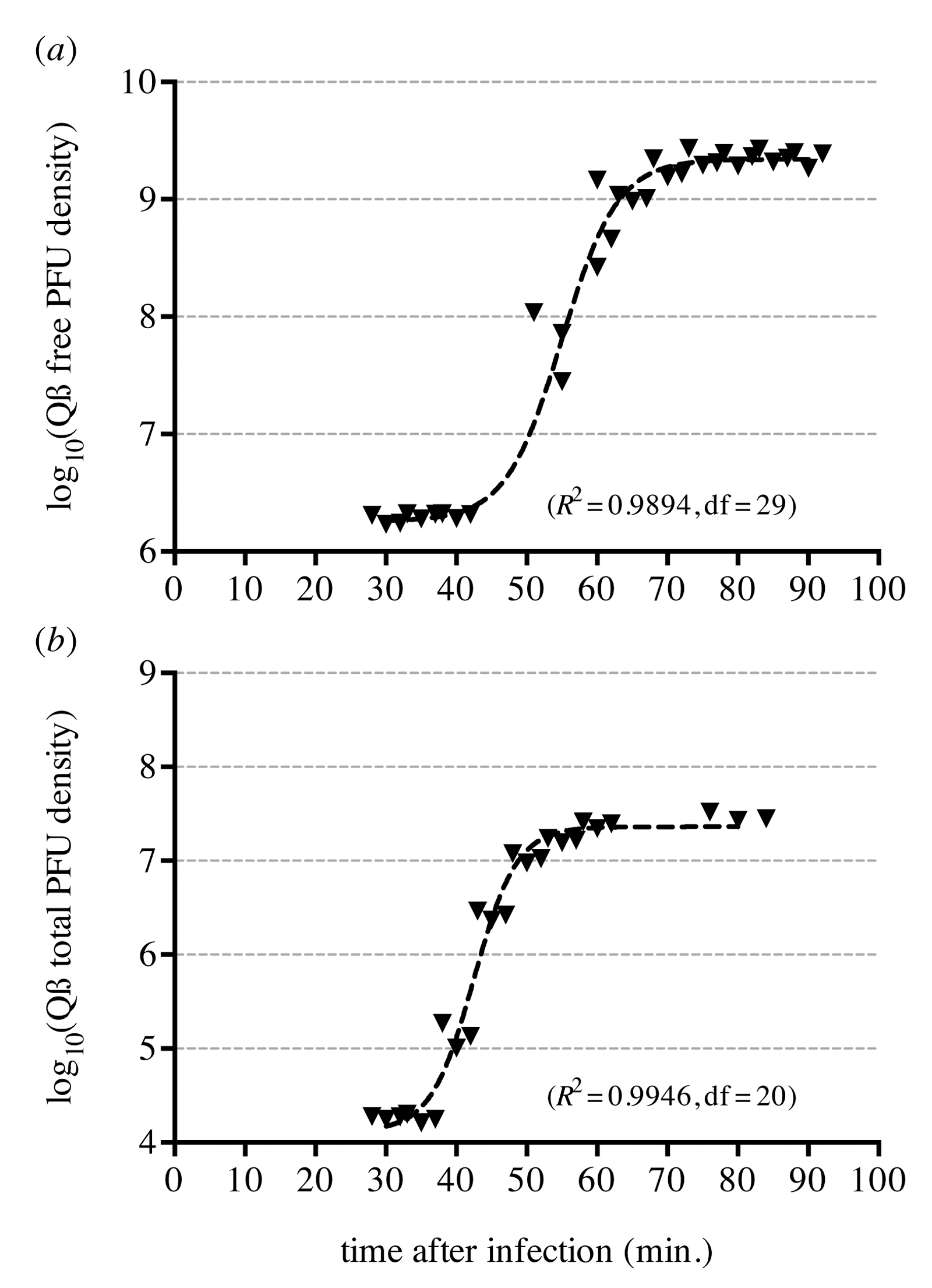

Supplement: García-Villada & Drake_ESM [file rsob130043-s1.docx]
